# Rhabdomyolysis associated with concomitant use of colchicine and statins in the real world: identifying the likelihood of drug–drug interactions through the FDA adverse event reporting system

**DOI:** 10.3389/fphar.2024.1445324

**Published:** 2024-09-16

**Authors:** Sha Zhang, Ming-Ming Yan, Hui Zhao, Xiao-Yan Qiu, Deqiu Zhu

**Affiliations:** ^1^ Department of Pharmacy, Tongji Hospital, School of Medicine, Tongji University, Shanghai, China; ^2^ Department of Pharmacy, Huashan Hospital, Fudan University, Shanghai, China

**Keywords:** colchicine, statins, rhabdomyolysis, drug-drug interactions, pharmacovigilance

## Abstract

**Background:**

Currently, there remains substantial controversy in research regarding whether the concomitant use of colchicine and statins increases the occurrence of rhabdomyolysis, warranting further substantiation.

**Objective:**

This study aimed to identify the likelihood drug-drug interactions (DDIs) for the co-administration of colchicine and statins resulting in rhabdomyolysis.

**Methods:**

A disproportionality analysis was conducted by using data sourced from the US Food and Drug Administration Adverse Event Reporting System (FAERS) to detect rhabdomyolysis signals associated with the combined use of colchicine and statins. The association between (colchicine/statins/colchicine and statins) and rhabdomyolysis were evaluated using information component (IC). DDI signals were calculated based on the Ω shrinkage measure and Bayesian confidence propagation neural network (BCPNN) method. Furthermore, stratification was performed based on colchicine and individual statins agents.

**Results:**

In total, 11,119 reports of rhabdomyolysis were identified in the FAERS database, 255 (2.29%) involved both colchicine and statins. Our analysis showed potential DDI signals of rhabdomyolysis (Ω_025_ = 1.17) among individuals concurrent use of colchicine and statins. Moreover, further drug-specific analysis suggests DDI signals in the colchicine-atorvastatin pair (Ω_025_ = 1.12), and colchicine-rosuvastatin pair (Ω_025_ = 1.05), along with a higher proportion of rhabdomyolysis (IC_025_ = 5.20) and (IC_025_ = 4.26), respectively.

**Conclusion:**

The findings suggest that concomitant use of colchicine and statins may increase the risk of rhabdomyolysis, particularly when combined with atorvastatin or rosuvastatin. Therefore, healthcare professionals should pay special attention to life-threatening AE such as rhabdomyolysis, when co-prescribing colchicine statins.

## 1 Introduction

Statins, also known as 3-hydroxy-3-methyl-glutaryl coenzyme A reductase inhibitors, are used to reduce cholesterol and triglycerides by blocking the formation of cholesterol in the liver (Fiolet et al., 2021). Statins have been a cornerstone class of medications in cardiovascular therapeutics for decades, owing to their lipid-lowering and anti-inflammatory benefits as well as their role in the primary and secondary prevention of cardiovascular disease (Grundy et al., 2004).

Colchicine, a traditional anti-inflammatory medication, has been used to treat and prevent gout flares for millennia. In the last decade, large placebo-controlled trials in nearly 12,000 patients have confirmed that colchicine significantly reduces the risk of myocardial infarction, ischemic stroke, and the need for unscheduled revascularization by 25%–30% based on anti-platelet and lipid-lowering therapy (Turner and Pirmohamed, 2019; Siak et al., 2021). Thus, the US Food and Drug Administration (FDA) approved colchicine (Sadiq et al., 2024) in 2023 for patients with atherosclerotic disease or multiple cardiovascular disease risk factors to reduce the risk of myocardial infarction, stroke, coronary revascularization, and cardiovascular death (Aimo et al., 2021). Evidence suggests that gout is independently linked to an increased risk of cardiovascular diseases (Choi and Curhan, 2007; Mouradjian et al., 2020). As the high and direct correlation between gout and cardiovascular disease, colchicine is extensively used in those patients (Tardif et al., 2019; Kaul et al., 2021). Accordingly, colchicine and statins are frequently co-prescribed for prevention and treatment of cardiovascular diseases, auto-inflammatory diseases, and gout, which is high and clinically significant.

Despite the benefits, both statins and colchicine are known to be associated with an increased risk of myotoxicity, ranging from mild myalgia to rhabdomyolysis, are major side effects and a leading cause of statin intolerance. While the precise incidence of myotoxicity induced by colchicine remains uncertain, statins-associated myotoxicity occurs in 5%–10% of cases, with rhabdomyolysis occurring in 0.01%–0.1% (Ramachandran and Wierzbicki, 2017; Newman et al., 2019). Rhabdomyolysis is the most serious side effect of statins, which leads to a high mortality rate of approximately 10% (Zutt et al., 2014; Toth et al., 2018; Fracchiolla et al., 2009). Therefore, there is a growing concern about the association between the combined use of colchicine and statins and the occurrence of myotoxicity, but this remains controversial. Several clinical studies have confirmed that long use of colchicine based on statins therapy does not increase the risk of myotoxicity and rhabdomyolysis (Wiggins et al., 2016; Kwon et al., 2017; Vrachatis et al., 2021). Conversely, other studies show an association between colchicine use and the occurrence of statins-related myotoxicity (Wiggins et al., 2016; Sen et al., 2021; Schwier et al., 2022).

The inconsistent results surrounding this issue remain controversial, thus, necessitating further clarification regarding whether the concurrent administration of colchicine and statins enhances the likelihood of myotoxicity, particularly rhabdomyolysis. Clinical trials and foundational research play a pivotal role in elucidating the mechanisms of action and the resultant effects of medications (Massari et al., 2020). On the other hand, real-world research holds significance in post-marketing drug safety monitoring and guiding clinical practice (Meng et al., 2022). Importantly, analyses combined with pharmacovigilance data could provide valuable evidential support. To enhance these endeavors, the FDA launched the US FDA Adverse Event Reporting System (FAERS) database to meticulously monitor the safety profile of approved pharmaceutical products. Data mining from the FAERS dataset can be employed for quantitative assessment in detecting drug-drug interactions (DDIs).

This study aimed to clarify whether concomitant use of statins and colchicine increases the incidence of rhabdomyolysis, thereby effectively addressing the limitations of previous studies.

## 2 Methods

### 2.1 Data acquisition and preprocessing

**FIGURE 1:**
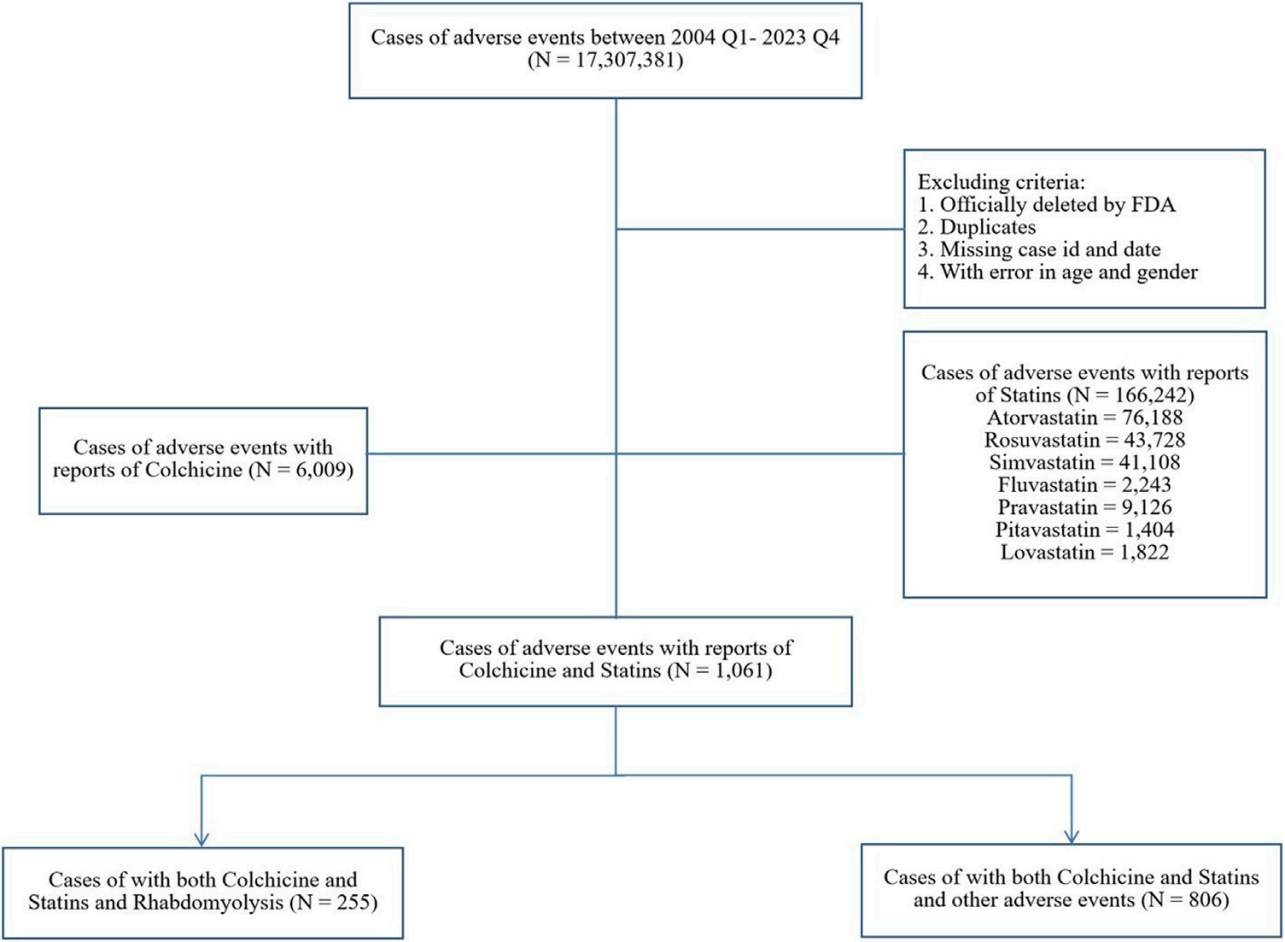
Data flow chart.

FAERS is a publicly available database that facilitates the surveillance of post-marketing safety for approved medications and biologics (Fukazawa et al., 2018). As an open-access platform, it proactively encourages adverse events (AEs) reporting via the MedWatch program, including consumers, healthcare professionals, pharmaceutical companies, and the general public (Meng et al., 2022). In our study, publicly available FAERS data from quarter 1 of 2004 to quarter 4 of 2023 were downloaded as raw data. The exclusion criteria were outlined in the study schematic (Figure 1): reports officially removed by FDA authorities, duplicates, those lacking case ID or date, and those containing inaccurate gender or age data were excluded. Ethical approval was not required as only anonymous data was used.

### 2.2 Identification of adverse events

In the FAERS database, AEs were coded using Preferred Terms from the Medical Dictionary for Regulatory Activities (MedDRA, version 26). AEs of myotoxicity were identified using the preferred term “rhabdomyolysis (Code10039020)” in MedDRA. Statins were defined as the following drugs: atorvastatin, rosuvastatin, fluvastatin, simvastatin, pravastatin, pitavastatin, and lovastatin, which are all listed in the FAERS. All reports referring to the trade name and generic name of the interested drugs were extracted for further analysis according to the scheme of study, and cleaned by the including or excluding criteria.

### 2.3 Descriptive analysis

Descriptive analysis on the demographic profiles was determined based on patients’ demographic. Categorical variables are presented with case number (N) and frequencies (N %), while continuous variables are presented using mean and medians with quartile ranges.

### 2.4 Disproportionality analysis

A sequential methodology was adopted to mitigate significant confounding variables and biases, adhering to the principles of Good Signal Detection Practices in Pharmacovigilance (Khaleel et al., 2022).

1) The disproportionality analysis based on differences in reporting portion of AEs. If there is no causal relationship between target drug and interested AE, the reporting rate will be similar to the average reporting rate for all other drugs, otherwise the associated drug-AE combination will demonstrate high reporting rate than random drug-AE pairs. Herein, we did not apply restrictions to the role code of each drug as either primary suspect or secondary suspect, as our aim was to analyze DDIs. As a result, all drugs were included in the study. Three mutually exclusive datasets were categorized: reports of colchicine alone, reports of statins alone, as well as reports with both colchicine and statins (colchicine + statins). In addition, we further refined the colchicine and statins dataset to focus specifically on colchicine in combination with individual statins. A disproportionality analysis was performed to compare these three datasets with all other drugs reported in the FAERS database by using the Bayesian confidence propagation neural network (BCPNN) method. This approach is more accurate than the Reporting Odds Ratio (ROR), particularly in situations with a limited number of cases (Noren et al., 2013). A reporting signal was defined as the lower limit of the 95% confidence interval (CI) of information component (IC_025_) > 0. The higher the IC_025_, the stronger signal between the drug and AE. The calculation procedure for the IC can be referenced in Supplementary Table S1.

2) To investigate the potential impact on the disproportionate reporting of rhabdomyolysis associated with co-administration of statins and colchicine by gender and age, we conducted a subgroup analysis by stratifying patients based on gender and age.

### 2.5 Drug-drug interaction analysis

A DDI analysis were conducted between colchicine and statins class level and specific-statins level by using BCPNN method and Ω shrinkage measure. We compared the IC_025_ values of colchicine and statins administered alone versus in combination, when the IC_025_ for the colchicine and statins combination exceeds that of other non-combination groups, it indicates evidence of interaction (IC_025_ of combination drugs greater than IC_025_ of individual drugs used). Additionally, to test the consistency of the findings, we further measured the DDI signals using the Ω shrinkage recommended by the World Health Organization Uppsala Monitoring Center, as an earlier study (Noguchi et al., 2020) showed it to be the most conservative of the multiple algorithms. Norén et al. (Noguchi et al., 2021) proposed the Ω shrinkage measure to calculate the observed-to-expected ratio as a measure of disproportionality to explore signals of DDIs. The detection criterion is the lower limit of the 95% CI of the Ω (Ω_025_) > 0. Consequently, the DDI signals detection criterion in our study is the lower limit of the 95% CI of the Ω (Ω_025_) > 0. The calculation methodology for Ω were elucidated in Supplementary Table S2. Therefore, the DDI signals detection criteria in this study require simultaneous meeting of the standards of both detection methods mentioned above.

### 2.6 Sensitivity analyses

To test the robustness of the DDI signals, we performed a sensitivity analysis using concomitant use of atorvastatin and clarithromycin as the positive control, in which we replicated our primary analysis using an established cytochrome p450-mediated DDIs between statins and clarithromycin to evaluate our ability to detect interaction-positive signals (Alkabbani et al., 2022; Chuma et al., 2022). In addition, we repeated our analysis with concomitant use of colchicine and aspirin as a negative control group, because the package-inserts of aspirin and previous studies (Wiggins et al., 2016) showed that there is no DDI between statins and aspirin. All Data processing and analysis were conducted using Microsoft Office Excel (2010) and SPSS (version 22), while R (4.12) was applied for graphics.

## 3 Results

### 3.1 Descriptive analysis

From quarter 1 of 2004 to quarter 4 of 2023, a total of 17,307,381 reports were retrieved through FAERS after data cleaning, among which 11,119 reports were associated with rhabdomyolysis (Figure 1). Of these, 255 (2.29%) involved both colchicine and statins, and the most common statin was atorvastatin (N = 140, 54.90%). The distribution of reported cases shows a notable upward trend over the years, primarily submitted by healthcare professionals. Specifically, reports involving colchicine alone, statins alone, or a combination of both were reported by healthcare professionals at rates of 88.76%, 89.81%, and 94.51%, respectively. For the combination therapy, the average age was 66.32 ± 13.31, with 75.29% being male patients, about three times as many as female. A significant portion of AE reports related to rhabdomyolysis were severe, leading to hospitalization (85.49%), life-threatening conditions (12.55%), or even death (11.37%) (Table 1).

**TABLE 1:** Demographic characteristics of cases of rhabdomyolysis.

Characteristic	Rhabdomyolysis		
	Colchicine alone, N (%) N = 267	Statin alone, N (%) N = 10,597	Colchicine and statin, N (%) N = 255
Age in years, mean (SD)	55.58 ± 18.62	66.64 ± 13.30	66.32 ± 13.31
Age in years, median(IQR)	59.00 (43.00,70.00)	68.00 (58.00,76.00)	69.00 (61.00, 75.00)
Gender			
Male	202 (75.66)	6,617 (62.44)	192 (75.29)
Female	65 (24.34)	3,977 (37.53)	63 (24.71)
Other	NA	3 (0.03)	NA
Colchicine n (%)	267 (100.00)	NA	255 (100.00)
Statins, n (%)	NA	10,597 (100.00)	255 (100.00)
Atorvastatin	NA	3,498 (33.01)	140 (54.90)
Rosuvastatin	NA	2,263 (21.36)	51 (20.00)
Simvastatin	NA	4,266 (40.26)	62 (24.31)
Fluvastatin	NA	173 (1.63)	9 (3.53)
Pravastatin	NA	363 (3.43)	12 (4.71)
Lovastatin	NA	155 (1.46)	2 (0.78)
Year			
2004	7 (2.62)	961 (9.07)	10 (3.92)
2005	7 (2.62)	962 (9.08)	6 (2.35)
2006	3 (1.12)	699 (6.60)	8 (3.14)
2007	3 (1.12)	585 (5.52)	9 (3.53)
2008	7 (2.62)	625 (5.90)	10 (3.92)
2009	15 (5.62)	716 (6.76)	6 (2.35)
2010	16 (5.99)	903 (8.52)	21 (8.24)
2011	28 (10.49)	985 (9.30)	19 (7.45)
2012	11 (4.12)	675 (6.37)	13 (5.10)
2013	13 (4.87)	538 (5.08)	8 (3.14)
2014	10 (3.75)	457 (4.31)	15 (5.88)
2015	7 (2.62)	445 (4.20)	1 (0.39)
2016	23 (8.61)	401 (3.78)	27 (10.59)
2017	16 (5.99)	409 (3.86)	13 (5.10)
2018	11 (4.12)	866 (8.17)	17 (6.67)
2019	18 (6.74)	844 (7.96)	28 (10.98)
2020	17 (6.37)	873 (8.24)	31 (12.16)
2021	39 (14.61)	695 (6.56)	29 (11.37)
2022	30 (11.24)	545 (5.14)	17 (6.67)
2023	20 (7.49)	609 (5.75)	10 (3.92)
Time to onset			
instant	11 (4.12)	255 (2.41)	2 (0.78)
1–2 days	3 (1.12)	47 (0.44)	NA
2–3 days	2 (0.75)	35 (0.33)	NA
3 days–1 week	6 (2.25)	113 (1.07)	NA
1–2 weeks	6 (2.25)	165 (1.56)	NA
0.5–1 month	19 (7.12)	372 (3.51)	7 (2.75)
1–3 months	6 (2.25)	723 (6.82)	12 (4.71)
3–6 months	2 (0.75)	372 (3.51)	3 (1.18)
0.5–1 year	3 (1.12)	363 (3.43)	5 (1.96)
1–2 years	NA	388 (3.66)	6 (2.35)
2–3 years	NA	236 (2.23)	1 (0.39)
3–4 years	1 (0.37)	166 (1.57)	NA
4–5 years	NA	111 (1.05)	3 (1.18)
5–10 years	4 (1.50)	248 (2.34)	6 (2.35)
TTO mean (SD) (day)	205.46 ± 622.41	560.72 ± 1062.25	578.42 ± 882.95
TTO median(IQR) (day)	21.00 (2.50, 28.00)	119.00 (27.00,647.00)	129.00 (40.00,555.00)
Reporter			
Health professional	237 (88.76)	9,517 (89.81)	241 (94.51)
Nonhealth professional	11 (4.12)	736 (6.94)	5 (1.96)
Unknown or missing	19 (7.12)	344 (3.25)	9 (3.53)
Outcomes			
Hospitalization/hospitalization prolonged	216 (80.90)	8,109 (76.52)	218 (85.49)
Other serious illness	136 (50.94)	4,921 (46.42)	113 (44.31)
Death	57 (21.35)	1,043 (9.84)	29 (11.37)
Life-threatening	26 (9.74)	1995 (18.83)	32 (12.55)
Requair medical intervention	7 (2.62)	389 (3.67)	8 (3.14)
Disability	3 (1.12)	864 (8.15)	11 (4.31)
Other	NA	92 (0.82)	NA
Occurred countries			
United States	49 (18.35)	1,058 (9.98)	47 (18.43)
France	47 (17.60)	845 (7.97)	64 (25.09)
Italy	9 (3.37)	521 (4.92)	20 (7.84)
Canada	11 (4.12)	287 (2.71)	7 (2.75)
Portugal	7 (2.62)	82 (0.77)	7 (2.75)
China	7 (2.62)	142 (1.34)	1 (0.39)
Turk	7 (2.62)	10 (0.09)	1 (0.39)
Missing	130 (48.7)	7,652 (72.2)	108 (42.4)

SD, standard deviation; IQR, interquartile range; NA, not applicable; TTO, Time-to-onset. The onset time was calculated as (onset date of AE)—(administration start date).

### 3.2 Disproportionality analysis

**FIGURE 2:**
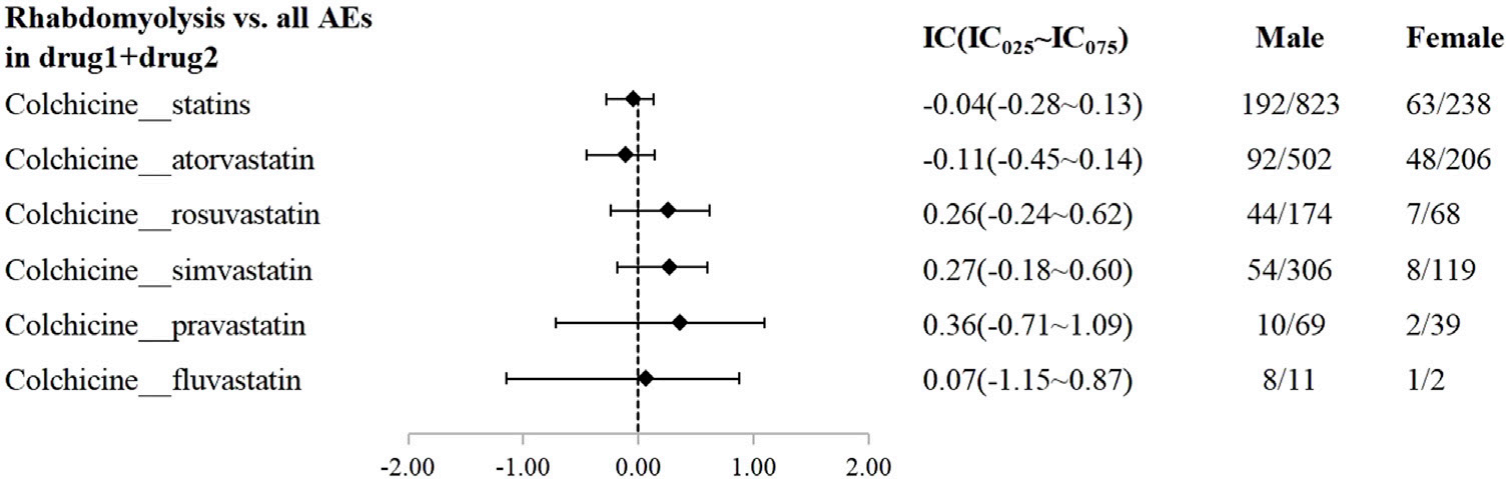
The Effect of gender on rhabdomyolysis reports with colchicine and statins. When IC025 > 0, a significant signal of rhabdomyolysis associated with combination therapy was detected between genders, with a higher risk in males compared to females.

**FIGURE 3:**
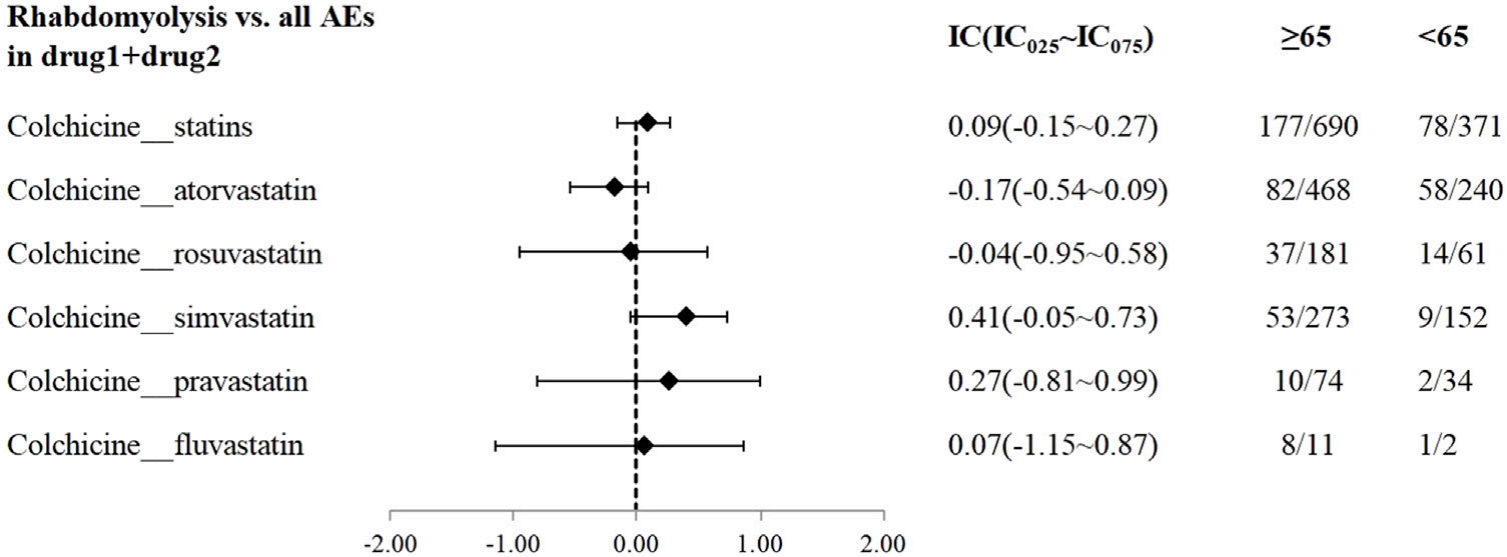
The Effect of age on rhabdomyolysis reports with colchicine and statins. When IC025 > 0, a significant association between age and the risk of rhabdomyolysis related to combination therapy was observed, with a higher risk in individuals aged ≥65 years compared to those <65 years.

A disproportionality analysis was conducted by comparing the three datasets with all other drugs reported in the FAERS database. Colchicine (IC_025_ = 3.68) or statins (IC_025_ = 4.43) alone both showed a signal of rhabdomyolysis. Among the included statins, including atorvastatin (IC_025_ = 3.94), simvastatin (IC_025_ = 5.11), rosuvastatin (IC_025_ = 4.09), and pravastatin (IC_025_ = 3.58), fluvastatin (IC_025_ = 4.29), and lovastatin (IC_025_ = 4.37), presented a disproportionate signal of rhabdomyolysis. Similarly, the concomitant use of colchicine and statins suggested a high-intensity rhabdomyolysis signal (IC_025_ = 5.73). Regarding colchicine-specific statins, including colchicine-atorvastatin, colchicine-rosuvastatin, colchicine-simvastatin, and colchicine-pravastatin, there are indications of rhabdomyolysis signals with signal strengths of IC025 = 5.20, 4.29, 4.26, and 1.63, respectively (Table 2). On performing gender and age stratification, we did not detect significant differences between males and females on the safety signals for the combination of colchicine and statins leading to rhabdomyolysis, either at the class level (IC_025_ = −0.28) or for specific drugs (Figure 2). Likewise, we did not find a significant difference in rhabdomyolysis signals between individuals more than 65 years and those under 65 years when colchicine was used in combination with statins (IC_025_ = −0.15) (Figure 3).

**TABLE 2:** IC_025_ and Ω_025_ of Colchicine and statins.

Drug1	Drug2	N of rhabdomyolysis with drug1+drug2/N of all AEs with drug1+drug2	Drug1+Drug2 IC(IC_025_-IC_075_)	Ω(Ω025-Ω075)	N of rhabdomyolysis with drug1/N of all AEs with drug1	Drug1 IC(IC_025_-IC_075_)	N of rhabdomyolysis with drug2/N of all AEs with drug2	Drug2 IC(IC_025_-IC_075_)
Colchicine	Statins	255/1,061	5.98 (5.73–6.22)	1.24 (1.17–1.32)	267/6,009	3.87 (3.68–4.06)	10,597/166,242	4.46 (4.43–4.49)
Colchicine	Atorvastatin	140/708	5.53 (5.20–5.87)	1.22 (1.12–1.32)	267/6,009	3.87 (3.68–4.06)	3,498/76,188	3.99 (3.94–4.04)
Colchicine	Simvastatin	62/426	4.82 (4.29–5.35)	0.08 (-0.08–0.23)	267/6,009	3.87 (3.68–4.06)	4,266/41,108	5.16 (5.11–5.21)
Colchicine	Rosuvastatin	51/242	4.93 (4.26–5.61)	1.21 (1.05–1.38)	267/6,009	3.87 (3.68–4.06)	2,263/43,728	4.16 (4.09–4.22)
Colchicine	Pravastatin	12/108	3.30 (1.63–4.98)	0.48 (0.13–0.83)	267/6,009	3.87 (3.68–4.06)	363/9,126	3.74 (3.58–3.90)
Colchicine	Fluvastatin	9/13	3.26 (-1.37–7.89)	2.27 (1.87–2.67)	267/6,009	3.87 (3.68–4.06)	173/2,243	4.54 (4.29–4.79)

When IC_025_ > 0, a significant signal was detected between target drug and rhabdomyolysis; When Ω_025_ > 0,and (drug1+drug2 IC_025_)> (drug1C_025_) and (drug2C_025_), a significant drug–drug interaction signal was detected.; IC, information component; AEs, Adverse events.

### 3.3 Drug-drug interactions

A DDI analysis were conducted between colchicine and statins associated with rhabdomyolysis. We observed a heightened disproportionality in the reporting of rhabdomyolysis with concomitant administration of colchicine and statins, evidenced by the IC_025_ value (IC_025_ = 5.73) for the combination exceeding that of the individual drugs (IC_025_ = 3.68 and IC_025_ = 4.43). Meanwhile, the Ω shrinkage model (Ω_025_ = 1.17) identified a DDI signal when colchicine was co-treated with statins, indicating an elevated risk of rhabdomyolysis (Table 2).

In addition, we carried out this possible DDI on a drug-specific level between colchicine and statins. Concerning drug-specific results, colchicine-atorvastatin pair and colchicine-rosuvastatin pair demonstrated DDI signals of rhabdomyolysis in both the Ω shrinkage model (Ω_025_ = 1.12 and Ω_025_ = 1.05) and BCPNN method (IC_025_ = 5.20 and IC_025_ = 4.26). However, significant DDI signals of rhabdomyolysis were observed for colchicine-pravastatin pair and colchicine-fluvastatin pair in the Ω shrinkage model (Ω_025_ = 0.13 and Ω_025_ = 1.87), but they were absent in the BCPNN method. And the combined treatment cases of colchicine and fluvastatin are limited, comprising only 13 cases, including 9 cases and 4 non-cases. Furthermore, regarding colchicine and simvastatin, there was no evidence of a significant interaction from the Ω-shrinkage model, but it was at the edge of the threshold (Ω_025_ = −0.08). (Table 2).

### 3.4 Sensitivity analyses

Two pre-specified sensitivity analyses were conducted, encompassing positive control analysis and negative control analysis. In the positive control analysis establishing DDI signals between clarithromycin and simvastatin, both estimations including the Ω-shrinkage model (Ω_025_ = 0.84) and BCPNN method (IC_025_ = 5.61, greater than IC_025_ of individual drugs used) indicated significant DDI signals. Conversely, in the negative control analysis assessing the combined use of atorvastatin and aspirin, all estimations suggested the absence of a DDI (Ω_025_ = -0.39) and BCPNN method (IC_025_ = 3.44, less than IC_025_ of individual drugs used) (Table 3).

**TABLE 3:** IC_025_ and Ω_025_ of sensitivity analyses.

Drug1	Drug2	N of rhabdomyolysis with drug1+drug2/N of all AEs with drug1+drug2	Drug1+Drug2 IC(IC_025_-IC_075_)	Ω(Ω025-Ω075)	N of rhabdomyolysis with drug1/N of all AEs with drug1	Drug1 IC(IC_025_-IC_075_)	N of rhabdomyolysis with drug2/N of all AEs with drug2	Drug2 IC(IC_025_-IC_075_)
Clarithromycin	Simvastatin	239/1,084	5.86 (5.61–6.11)	0.91 (0.84–0.99)	343/18,506	2.66 (2.50–2.82)	4,266/41,108	5.16 (5.11–5.21)
Acetylsalicylic	Atorvastatin	244/6,465	3.64 (3.44–3.84)	−0.32 (-0.39-0.24)	95/25,539	0.36 (0.07–0.66)	3,498/76,188	3.99 (3.94–4.04)

When IC_025_ > 0, a significant signal was detected between target drug and rhabdomyolysis; When Ω_025_ > 0,and (drug1+drug2 IC_025_)> (drug1C_025_) and (drug2C_025_), a significant drug–drug interaction signal was detected. IC, information component; AEs, Adverse events.

## 4 Discussion

Current evidence from clinical research and case series studies does not conclude whether colchicine combined with statins therapy increases rhabdomyolysis (Aimo et al., 2021; Hansten et al., 2023). In this study, we performed a disproportionation analysis in tandem with BCPNN based on the large publicly available FAERS database to investigate possible DDI signals generated from combination therapy of colchicine and statins. We have four main findings: first, we confirmed signals of disproportionate reporting for rhabdomyolysis with colchicine or statins; second, we found gender did not exert a significant influence on the safety signal associated with the combination of colchicine and statins leading to rhabdomyolysis; third, we detected potential DDI signals between colchicine and statins that co-administration patterns increase the incidence of rhabdomyolysis; finally, we further identified potential DDI signals of rhabdomyolysis at a drug-specific level between colchicine and atorvastatin or rosuvastatin.

We carried out a disproportionality analysis of the three datasets, including colchicine, statins, and both colchicine and statins, against all other drugs in the FAERS database, and we found that colchicine or statins use alone had a clear potential signal for rhabdomyolysis. The findings regarding colchicine and statins align with existing literatures and labeling information, affirming the reliability of our study’s methodology and data analysis. Prior studies indicate a heightened risk of myotoxicity, notably rhabdomyolysis, among individuals using either colchicine or statins (Turner and Pirmohamed, 2019; Newman et al., 2019; Zutt et al., 2014). Furthermore, drug inserts underscore the importance of vigilance regarding the occurrence of myotoxic AEs during administration.

Statins are the cornerstone of treating cardiovascular diseases as well as metabolic disorders, more and more patients are being treated with statins in clinical practice (Wiggins et al., 2016). Given the growing interest in targeting inflammation to mitigate major cardiovascular risk and the significant anti-inflammatory effects of colchicine, along with its potential to reduce major cardiovascular events, related research is receiving increasing attention (Xie et al., 2024). This is further compounded by the FDA’s 2023 approval of colchicine for patients with atherosclerotic vascular disease or multiple cardiovascular risk factors, as extensive clinical studies over the past decade have validated its contribution in reducing cardiovascular events (Aimo et al., 2021; Mouradjian et al., 2020; Tardif et al., 2019). Therefore, the potential co-medication of colchicine and statins is high. We performed a co-administration analysis of colchicine and statins to investigate potential DDIs by BCPNN method and Ω shrinkage analysis. The results showed that there are potential DDI signals between colchicine and statins which increased the reported frequency of rhabdomyolysis. Our findings are consistent with a recent review reported by Schwier et al. (2022) which suggests that the statins-colchicine drug interaction may be related to potentially life-threatening myotoxicity. Upon performing gender stratification, we found that gender did not significantly influence the safety signal associated with the combination of colchicine and statins leading to rhabdomyolysis. Previous studies have highlighted gender as a significant risk factor for myopathy or rhabdomyolysis, with females exhibiting a higher frequency of reported myotoxicity compared to males (Ward et al., 2019; Mancini et al., 2016). Our results can be interpreted as the incidence of cardiovascular diseases being higher in males than females (Virani et al., 2023),as well as the number of individuals co-administering colchicine and statin being significantly greater in males. Specifically, in our study, the number of males combined with colchicine and statins was three times greater than that of females.

In the age-stratified analysis, this study found that the number of patients over 65 years using both colchicine and statins is twice that of those under 65 years old. This finding reflects the increasing use of both colchicine and statins among the elderly, consistent with longer life expectancy and rising rates of cardiovascular disease. Studies have shown that the elderly may be more susceptible to statins-induced myotoxicity, but specific age ranges have not been clearly defined (Adhyaru and Jacobson, 2018). However, this study found no significant difference in rhabdomyolysis signals between individuals over and under 65 years old when colchicine was combined with statins. This result aligns with Camerino et al. (2017) which indicated that in most subjects, modifications in skeletal muscle biomarkers due to statins therapy were independent of age. It is important to note that our result does not suggest that advanced age is not a critical factor. Statins-associated muscle toxicity is influenced by several factors, including high doses, advanced age, female sex, hypothyroidism, reduced muscle mass, and increased physical activity (Adhyaru and Jacobson, 2018). We hypothesize that the risk of myotoxicity may increase only when multiple complex adverse factors are present. Addressing one or more of these factors could reduce or eliminate the risk of muscle toxicity. Importantly, clinicians should carefully evaluate each patient’s condition and address factors contributing to statins-induced muscle toxicity to optimize the safety and efficacy of statins therapy. Besides, positive and negative controls were employed to assess both the internal validity of the database and the robustness of the DDI signals. The clarithromycin-simvastatin pair was utilized as the positive control, while the atorvastatin-aspirin pair served as the negative control. The outcomes pertaining to these pre-defined control drugs aligned with our expectations, thus reinforcing the reliability and validity of this study concerning both methodology and data analysis.

We conducted further analyses to explore potential DDIs specifically between colchicine and certain statins. Our drug-specific analysis revealed potential signals of DDIs leading to increase of drug-induced rhabdomyolysis due to co-administration of colchicine and atorvastatin. For many years, it has been posited that lipophilic statins, such as atorvastatin and simvastatin, are more prone to inducing muscle toxicity compared to hydrophilic statins like rosuvastatin and pravastatin (Sabanis et al., 2021). This is attributed to the fact that lipophilic statins are predominantly metabolized by the hepatic cytochrome P450 3A4 (CYP3A4) enzyme system, whereas hydrophilic statins are less reliant on CYP3A4 for their metabolism. Similarly, colchicine is lipophilicis and metabolized primarily by CYP3A4 and is eliminated via the P-glycoprotein pump, akin to lipophilic statins (Dahan et al., 2009; Finkelstein et al., 2010). The competition for the same metabolic enzymes and efflux pumps between colchicine and lipophilic statins may result in elevated plasma levels of these drugs (Davis and Wason, 2014). This is probably the most significant reason for the increase in adverse effects due to the combination of colchicine and statins. In addition, previous meta-analysis (Schwier et al., 2022) showed that among patients experiencing AEs due to the combination therapy of statins and colchicine, over 70% were attributed to atorvastatin or simvastatin, and simvastatin has the highest propensity for DDIs, especially in terms of pharmacokinetics (Siriangkhawut et al., 2017). Our study did not uncover a significant DDI signal between colchicine and simvastatin, but the results approached the threshold of significance. This suggested the necessity for additional research to delve deeper and corroborate these findings. However, it is important to emphasize that the absence of positive results does not necessarily imply the absence of rhabdomyolysis when colchicine and simvastatin are combined. Our research still indicated a strong signal for rhabdomyolysis with the concurrent use of these two drugs.

Our study also revealed potential DDI signals of rhabdomyolysis between colchicine and rosuvastatin, despite that rosuvastatin neither underwent metabolism via the CYP3A4 enzyme system nor being transported via the P-glycoprotein pathway. Another potential mechanism underlying the interaction between statins and colchicine may involve combined myotoxic effects, potentially additive or synergistic, given that each medication class has distinct mechanisms associated with causing myopathies (Ward et al., 2019; Fernandez-Cuadros et al., 2019; Wilbur and Makowsky, 2004; Camerino et al., 2021). Encouragingly, our findings were consistent with previous studies (Wiggins et al., 2016; Hansten et al., 2023), and indicate that the concomitant use of pravastatin and colchicine minimal increase in the risk of rhabdomyolysis. Considering the benefits of co-administration and potential DDIs that may result in severe adverse outcomes, we should select statins with fewer DDI in combination with colchicine to maximize effectiveness and minimize side effects. While our findings contribute evidence towards enhancing safety warnings for these medications, further preclinical and large-scale clinical studies are remained warranted.

Our study has some certain limitations. The FAERS database is a spontaneous and anonymous reporting system, making underreporting, overreporting, or missing information inevitable (Gravel et al., 2023); Second, reporting sources in the FAERS database are diverse, encompassing healthcare professionals and consumers. This heterogeneity can impact the data quality and result in the omission of crucial details regarding treatment-related myotoxicity, such as significant comorbidities and comorbid medications; Third, spontaneous reporting data suffer from inherent limitations including the inability to establish a causal relationship between myotoxicity and the combined colchicine and statin, as well as the failure to calculate the incidence rate of myotoxicity (Meng et al., 2022).

## 5 Conclusion

Our large-scale pharmacovigilance study indicates that concomitant use of colchicine and statins may increase the risk of rhabdomyolysis. Moreover, this interaction primarily pertains to colchicine and specific statins, including atorvastatin and rosuvastatin. Therefore, healthcare professionals should pay special attention to life-threatening AEs such as rhabdomyolysis, when co-prescribing colchicine and statins.

## Data Availability

Publicly available datasets were analyzed in this study. This data can be found here: https://fis.fda.gov/extensions/FPD-QDE-FAERS/FPD-QDE-FAERS.html.
