# Supplementary material for: Rhabdomyolysis associated with concomitant use of colchicine and statins in the real world: identifying the likelihood of drug–drug interactions through the FDA adverse event reporting system
Source: Front Pharmacol. 2024 Sep 16;15:1445324. doi: 10.3389/fphar.2024.1445324 (PMC11439674; doi:10.3389/fphar.2024.1445324)
Supplement: Supplementary file 1 [file Table1.DOCX]

Supplementary Material

**1. Calculation method of IC is as following:**

**Table S1**:2×2 contingency table for signal detection

|  | **Targeted AEs** | **Other AEs** | **Total** |
| --- | --- | --- | --- |
| **Targeted drugs** | N_11_ | N_10_ | N_1+_ |
| **Other drugs** | N_01_ | N_00_ | N_0+_ |
| **Total** | N_+1_ | N_+0_ | N_++_ |

The statistical formula is as follows to calculate IC,

$$\begin{aligned} IC=\log_{2}\left( \frac{N_{\mathrm{observed}}+ 0.5}{N_{\mathrm{expected}}+ 0.5} \right)\#\left( 1 \right) \end{aligned}$$

$$\begin{aligned} N_{\mathrm{expected}}=\frac{\left( N_{\mathrm{drug}}*N_{\mathrm{effect}} \right)}{N_{\mathrm{total}}}\#\left( 2 \right) \end{aligned}$$

$$\begin{aligned} \mathrm{IC}_{025}=\log_{2}\left( \frac{N_{\mathrm{observed}}+ 0.5}{N_{\mathrm{expected}}+ 0.5} \right)-3.3*\left( N_{\mathrm{observed}}+0.5 \right)^{-\frac{1}{2}}-2*\left( N_{\mathrm{observed}}+0.5 \right)^{-\frac{3}{2}}\#\left( 3 \right) \end{aligned}$$

$$\begin{aligned} \mathrm{IC}_{975}=\log_{2}\left( \frac{N_{\mathrm{observed}}+ 0.5}{N_{\mathrm{expected}}+ 0.5} \right)+2.4*\left( N_{\mathrm{observed}}+0.5 \right)^{-\frac{1}{2}}-0.5*\left( N_{\mathrm{observed}}+0.5 \right)^{-\frac{3}{2}}\#\left( 4 \right) \end{aligned}$$

N_expected_: the number of case reports expected for the drug-adverse effect combination.

N_observed_: the actual number of case reports for the drug- adverse effect combination.(a)

N_drug_: the number of case reports for the drug, regardless of adverse effects.(a+b)

N_effect_: the number of case reports for the adverse effect, regardless of the drug.(a+c)

N_total_: the total number of case reports in the database. (a+b+c+d)

**2. Calculation of drug-drug interaction signal, Ω, is as following:**

**Table S2** The 4 × 2 contingency table for signal detection of drug-drug interaction.

|  | **Target AE** | **Other AEs** | **Total** |
| --- | --- | --- | --- |
| **Concomitant use of drug D1 and drug D2** | n111 | n110 | n11+ |
| **drug D1 without drug D2** | n101 | n100 | n10+ |
| **drug D2 without drug D1** | n011 | n010 | n01+ |
| **Neither drug D1 nor drug D2** | n001 | n000 | n00+ |
| **Total** | n++1 | n++0 | n+++ |

AE: adverse event, *n*: the number of reports.

$$\Omega={log}_{2}\frac{n_{111}+0.5}{E_{111}+0.5} \ldots\left( 5 \right)$$

$$f_{00}=\frac{n_{001}}{n_{00+}}, f_{10}=\frac{n_{101}}{n_{10+}}, f_{01}=\frac{n_{011}}{n_{01+}}, f_{11}=\frac{n_{111}}{n_{11+}} \ldots\left( 6 \right)$$

Where, *n* is the number of reports shown in the 4 × 2 contingency table.

$$g_{11}=1-\frac{1}{\max\left( \frac{f_{00}}{1-f_{00}}, \frac{f_{10}}{1-f_{10}} \right)+ \max\left( \frac{f_{00}}{1-f_{00}}, \frac{f_{01}}{1-f_{01}} \right)- \frac{f_{00}}{1-f_{00}} +1} \ldots\left( 7 \right)$$

When *f*_10_ < *f*_00_ (which denote no risk of AE caused by *drug D*_1_), the most sensible estimator *g*_11_ = max (*f*_00_, *f*_01_) is yielded and the *vice versa* when *f*_01_ < *f*_00_.

$$E_{111}=g_{11}\times n_{11+} \ldots(10)$$

$$\mathrm{Var}\left( \Omega_{0} \right)=\mathrm{Var}\left( {log}_{2}\frac{n_{111}}{E_{111}} \right)\approx\frac{1}{n_{111}{\log\left( 2 \right)}^{2}} \ldots(8)$$

Where, *n*_111_ is the number of reports and *E*_111_ is the expected value.

$$\Omega_{025}=\Omega-\frac{\phi\left( 0.975 \right)}{ln(2)\sqrt{n_{111}}} \ldots(9)$$

Where, *ϕ* (0.975) is 97.5% of the standard normal distribution.
